# Impact of distance education on primary health care indicators in central Brazil: An ecological study with time trend analysis

**DOI:** 10.1371/journal.pone.0214485

**Published:** 2019-03-26

**Authors:** Mara Lisiane Moraes dos Santos, Edilson José Zafalon, Rafael Aiello Bomfim, Vera Lucia Kodjaoglanian, Silvia Helena Mendonça de Moraes, Debora Dupas Gonçalves do Nascimento, Carlos Antonio de Souza Telles Santos, Albert Schiaveto de Souza, Alessandro Diogo De-Carli

**Affiliations:** 1 Integrated Health Institute, Federal University of Mato Grosso do Sul, Post graduate program stricto sensu in Family Health, Campo Grande, Mato Grosso do Sul, Brazil; 2 Faculty of Dentistry, Federal University of Mato Grosso do Sul, Campo Grande, Mato Grosso do Sul, Brazil; 3 Post-Doctoral researcher at Public Health School–University of São Paulo, São Paulo, Brazil; 4 Faculty of Dentistry, Federal University of Mato Grosso do Sul, Professor at Post graduate program stricto sensu in Family Health and stricto sensu in Dentistry, Campo Grande, Mato Grosso do Sul, Brazil; 5 State Secretary of Health, Mato Grosso do Sul, Brazil; 6 Researcher in Public Health, MSc, Oswaldo Cruz Foundation, Campo Grande, Mato Grosso do Sul, Brazil; 7 Researcher in Public Health, Instituto Gonçalo Muniz- Fiocruz, Salvador, Brazil; 8 Biosciences Institute, Federal University of Mato Grosso do Sul, Post graduate program stricto sensu in Family Health, Campo Grande, Mato Grosso do Sul, Brazil; Aga Khan University, KENYA

## Abstract

**Objective:**

The objective of this study was to verify whether the inclusion of professionals who completed a specialized distance learning course in family health teams is associated with rates of hospitalization for primary healthcare-sensitive conditions and better monitoring of chronic conditions in municipalities within the state of Mato Grosso do Sul, Brazil.

**Methods:**

Negative binomial regression models with fixed effects were used for the 79 municipalities in the state, with repeated observations for the selected years (2009–2015). For our reference, the parameter “Municipality Ratio” was the number of professionals who completed the course divided by the total number of PHC professionals in the municipality. This ratio has been cumulative over the years. No reference values were found in the scientific literature, so three cutoff points were used for tertile distribution: T3:high (0.35–1.00), T2:intermediate (0.02–0.33), and T1:Low (0.00–0.01). In order to avoid capturing biased results, the analysis was also performed for the years before the specialization course was offered (2009 and 2010).

**Results:**

Indicators of the share of hospitalizations for primary care-sensitive conditions (overall rate and specific rates for asthma, gastroenteritis, and heart failure) decreased during the study period when related to a high and intermediate proportion of professionals who completed the specialization course, and the same was seen for indicators of chronic conditions (diabetic and hypertensive patients) who were registered, monitored and group care.

**Conclusion:**

The specialization course impacted important indicators related to the attributions of primary health care professionals, considering that decreases in hospitalizations for primary care sensitive causes (overall rate of sensitive causes, specific rates for asthma, gastroenteritis and heart failure) were seen in the territories where professionals who completed this course worked, along with increased registration and monitoring of diabetic and hypertensive patients.

## Introduction

The Brazilian Unified Heath System (*Sistema Único de Saúde*, SUS) can be considered the world’s largest free and universal public health system [1]. Since its creation in 1990, access to health care has broadened significantly and there have been major movements to redirect the health care model, namely through implementing primary health care (PHC).

PHC is currently implemented primarily via the Family Health Strategy (FHS), a Brazilian state policy considered to be the heart of SUS [1,2]. The most elemental structure of FHS teams includes doctors, nurses, and community health agents, and the teams are organized geographically to care for up to 3500 people by team [3]. Each team member has attributions outlining practice responsibility, practice guidelines, and help the family health system responds to the majority of health problems [4]. The number of teams has risen remarkably since their implementation, from 2,000 teams providing services to 7 million people (4% of Brazil’s population) in 1998 to 43,000 teams that incorporate more than 700,000 workers and serve more than 134 million people (64.7% of the population) in 2018 [5].

There is strong evidence of the impact of FHS on important health indicators: decrease of infant mortality [1,6,7,8,9], hospitalizations due to causes sensitive to primary care [1,10], hospitalization and mortality rates due to cerebrovascular and cardiac diseases [11], health inequities [12], number of hospitalizations for heart failure and stroke [13], probability of reporting the emergency room or hospital as a usual source of care, and in cases in which the population declares one or more chronic illness, indicates the FHT as the usual source of care in the same proportion of the Brazilian population covered by a private plan (80%) [14].

However, despite the increased number of teams and the expansion of actions and services offered by FHS, the country faces problems implementing primary care, and the quality and effectiveness of the services offered must be improved. The quality of services varies, depending on the differences in the availability of basic equipment, variation of the profile of professionals, gaps in the coordination of care, insufficient specialized care services, different management models [15], as well as problems related to infrastructure, availability of inputs and professional qualification [16].

One of the major obstacles to FHS is the training of health workers in Brazil, which still has room for improvement in terms of the professional profile required to consolidate this strategy [17]. In spite of the important advances in the sector, there is misalignment between health training and the principles of SUS and PHC [18,19]. Professionals report that their main source of learning is in practice and in the reality of FHS, and have expressed their dissatisfaction with the academic training they received [20].

To address this training gap, the Brazilian Ministry of Health established the National Policy on Continuing Education (EPS) through the Department of Health Education and the Secretary of Labor Management and Health Education. This policy is intended to transform educational practices related to care, management, and organization of work in health through a training process which analyzes everyday work experiences. The EPS guidelines establish that regional characteristics should be considered in terms of health activities, training needs, and the capacity of formal institutions of health education. In this way, the policy stimulates partnerships with well-established educational institutions and decentralizes processes, which should specifically target the different practical realities of each region, for contextualized professional development. [21].

With the goal of expanding and catalyzing continuous education and training the labor force for SUS, in 2010 the Ministry of Health created the SUS Open University (UNA-SUS). The Objective of the program was to develop Continuous Professional Development (CPD) and training opportunities within the Brazilian workforce. This was achieved in 2010 through the formation of a collaborative network [22], so, specialization courses in Family Health were created, aimed to qualifying the PHC workers throughout the whole country, proposing large-scale training through distance education.

Within this context, the Oswaldo Cruz Foundation-Mato Grosso do Sul, the Federal University of Mato Grosso do Sul, and UNA-SUS established a specialization course in family health care (referred to here by the course’s Portuguese acronym, CEABSF) via distance education. The course was guided by the principles of PHC, enabling the development and strengthening professional skills, and to respond territory health needs. The purpose is qualify public health professionals with full graduation, who work in PHC in the state of Mato Grosso do Sul (MS), which is located in the midwest region of the country. The state was the first to develop FHC specialization via distance education successfully achieving implementation to 100% of municipalities.

The Ministry of Health funded the initiative as part of national policy. Measuring the impact of this specialization through distance education provides the basis for future national research [23], and, it is important to identify the impacts of this large-scale training process on health indicators related to quality and effectiveness of PHC services provided in the municipalities where CEABSF graduates work. Although many professionals have completed the specialization courses in family health through UNA-SUS, because it is a relatively recent initiative, no studies of the impacts these courses have on health indicators related to the effectiveness of PHC could be found in the literature. The course now has a significant level of graduates and sufficient numbers to investigate impact. The expectation is that better health indicators can be achieved through access to skilled care [19].

One indicator which is widely used to assess the effectiveness of PHC in different contexts is hospitalizations for Primary Care-sensitive Conditions (PCSC). This is considered a key indicator because it reveals important problems of access to the health system or its performance. These are hospitalizations that could have been avoided if the patient had had timely access to PHC to resolve the problem. In 2008, Brazil established a national list of these sensitive hospitalizations, which are an indicator of surveillance in health services and the performance of FHS in the country [23]. This work was developed by the Department of Basic Attention (DAB) of the Ministry of Health—composed of eight researchers with experience in primary health care and three members of the DAB technical team—who prepared the list based on literature review and with researchers and health managers help, the Brazilian Society of Family and Community Medicine, and Brazilian society in general [23]. The indicators of chronic conditions are also important in assessing the effectiveness of PHC, which total approximately 82% of health problems [24] and for the most part require longitudinal, coordinated care.

Despite abundant evidence of the benefits of strengthening PHC seen in different indicators of population health (both in low and mid [25], as well as in developed countries [26,27,28]), the relationship between health outcomes and the presence of professionals specialized in PHC in care teams has not been well studied. Studies investigating this relationship can support new formulations in policies related to training professionals for PHC, which will provide more effective services [19].

The objective of this study was to verify whether the presence of CEABSF graduates in FHS teams was associated with better rates of hospitalizations for primary care-sensitive conditions as well as better monitoring of chronic conditions in municipalities in the state of Mato Grosso do Sul, Brazil.

## Materials and methods

This study utilizes an ecological design with a temporal trend, with longitudinal data sets created for the years 2009–2015. The units of analysis were the 79 municipalities in the state of Mato Grosso do Sul.

The study considered coverage provided by professionals who completed the family health specialization course and served the population in PHC with a cumulative ratio over the period studied. The coverage of these professionals was established as the exposure variable for verification of improvements in PHC indicators, and the model was adjusted for fixed indexes (HDMI and Gini). This adjustment made it possible to obtain the effect on PHC indicators in relation to the proportion of professionals who completed the specialization course. For our reference, the parameter “Municipality Ratio” was the number of professionals who completed the course divided by the total number of PHC professionals in the municipality. This ratio has been cumulative over the years.

$$Municipality\;Ratio=\frac{number\;Professionals\;completed\;course\;in\;the\;city}{total\;number\;PHC\;professionals\;in\;the\;city}$$

No reference values were found in the previous scientific literature, so three cutoff points were used for tertile distribution: T_3_:high (0.35–1.00), T_2_:intermediate (0.02–0.33), and T_1_:Low (0.00–0.01). In order to avoid capturing biased results, the analysis was also performed for the years before the specialization course was offered (2009 and 2010).

In order to compare and validate our analysis, the effect of possible externalities (unrelated to the object of study), i.e., effects with positive repercussions on non-eligible inhabitants (with coverage by professionals who completed the course) in the municipality were also calculated. To do so, data on deaths resulting from traffic accidents, workplace accidents, and cancer were selected as indicators (Table 1, indicators 11, 12, and 13). Assessment of the data utilized a multidimensional validated criterion.

**Table 1:** Distribution of professional graduates and in PHC and health indicators (mean and CI95%) in Mato Grosso do Sul (2009–2015).

	2009	2010	2011	2012	2013	2014	2015
**Number of professional graduates/per city**	-	-	2.48	8.69	15.46	21.65	43.8
			(SD 4.28)	(SD 6.91)	(SD 9.06)	(SD 10.27)	(SD 36.39)
**Number of professional in primary care**	96.92	107.26	169.47	181.8	201.84	217.85	229.7
	(SD 255.60)	(SD 320.36)	(SD 468.29)	(SD 494.56)	(SD 597.82)	(SD 634.88)	(SD 706.31)
**Indicator 1**	10.84	12.17	10.02	10.22	8.45	7.97	7.62
**CI95%**	(9.23–12.46)	(10.49–13.84)	(8.50–11.54)	(8.62–11.81)	(7.11–9.79)	(6.77–9.18)	(6.40–8.84)
**Indicator 2**	3.4	2.88	2.49	1.84	1.57	1.36	1.21
**CI95%**	(2.58–4.21)	(2.14–3.63)	(1.80–3.17)	(1.40–2.28)	(1.24–1.91)	(1.08–1.65)	(0.95–1.48)
**Indicator 3**	2.28	1.84	1.8	1.71	1.4	1.54	1.21
**CI95%**	(1.75–2.81)	(1.44–2.24)	(1.46–2.15)	(1.31–2.11)	(1.05–1.74)	(1.12–1.96)	(0.94–1.48)
**Indicator 4**	43.33	42.77	42.35	41.71	39.37	39.93	38.09
**CI95%**	(40.53–46.13)	(40.04–45.49)	(39.55–45.14)	(38.93–44.49)	(36.90–41.83)	(37.65–42.21)	(35.82–40.35)
**Indicator 5**	2077.75	2122.92	2343.2	2516.65	3187.23	2229.17	2149.66
**CI95%**	(1216.14–2939.36)	(1280.80–2965.03)	(1463.95–3222.45)	(1491.22–3542. 08)	(1458.03–4916.43)	(1496.70–2961.65)	(1518.32–2780.99)
**Indicator 6**	7178.37	7129.16	12938.97	8036.53	7662.66	6703.97	6322.9
**CI95%**	(4746.26–9610.47)	(4819.62–9438.71)	(2470.41–23407.54)	(5394.01–10679.06)	(5007.97–10317.36)	(4848.25–8559.69)	(4486.50–8159.29)
**Indicator 7**	5394.93	5891.07	6303.11	8269.41	7009.92	5987.21	4743.24
**CI95%**	(1617.62–9172.24)	(1629.09–10153.06)	(1781.43–10824.79)	(2819.61–13719.20)	(2025.00–11994.84)	(1827.88–10146.55)	(2789.16–6697.32)
**Indicator 8**	4717.82	5049.88	5368.77	7231.78	5729.89	5115.23	4363.83
**CI95%**	(1875.66–7559.97)	(1939.42–8160.34)	(2049.61–8687.54)	(2745.68–11717.88)	(2244.71–9215.08)	(1961.36–8269.10)	(2729.05–5998.60)
**Indicator 9**	23507.38	24795.14	25488.4	28209.01	26240.47	22028.54	17324.13
**CI95%**	(9087.9–37926.8)	(8975.8–40614.5)	(9369.08–41607.7)	(11728.6–44689.4)	(9793.16–42687.8)	(8996.6–35060.4)	(11264.8–23383.5)
**Indicator 10**	20393.72	21082.12	21645.46	24187.15	21627.88	18682.12	15954.15
**CI95%**	(9819.9–30967.5)	(9851.6–32312.7)	(10120.2–33170.7)	(11993.1–36381.1)	(10394.7–32860.9)	(8848.2–28515.9)	(10813.2–21095)
**Indicator 11**	0.64	0.38	0.53	0.55	0.42	0.52	0.47
**CI95%**	(0.31–0.97)	(0.00–0.76)	(0.17–0.90)	(-0.05–1.16)	(-0.06–0.91)	(0.00–1.06)	(-0.01–0.95)
**Indicator 12**	30.21	26.21	30.34	26.97	30.57	32.33	32.71
**CI95%**	(8.37–52.06)	(6.03–46.40)	(7.24–53.45)	(6.52–47.42)	(7.37–53.77)	(6.69–57.97)	(7.69–57.74)
**Indicator 13**	9.97	8.7	12.25	8.29	10.23	10.57	8.38
**CI95%**	(4.38–15.56)	(3.22–14.18)	(5.31–19.20)	(3.99–12.58)	(4.93–15.52)	(5.01–16.13)	(3.49–13.27)
**State Gini**	0.52 (0.40–0.67)*						
**State Human Development Index**	0.67 (0.52–0.78)**						

*Gini Coefficient ranged from 0.40 to 0.67 in Mato Grosso do Sul municipalities in 2010. SD—Standard Deviation.

** HDI ranged from 0.52 to 0.78 in Mato Grosso do Sul municipalities in 2010. CI95%- Confidence interval.

The indicators which were selected and evaluated are related to PHC, and were consequently collected from the Basic Care Data System (SIAB), which was developed as a management instrument for local health systems, in addition to public data from the Brazilian Institute of Geography and Statistics [29].

Negative binomial regression models with fixed effects were used for the 79 municipalities in the state of Mato Grosso do Sul, with repeated observations for the selected years (2009–2015). Adjusting the binomial model for longitudinal data permits control of the characteristics of the analysis units (municipalities), which remain constant during the study period, while also allowing an estimate of the average strength of an association over the years analyzed for one indicator (response variable). The different indicators selected were analyzed by the measure of effect, relative risk (RR); this is easy to interpret by measuring the magnitude of the effect (values exceeding this unit indicate improvements in care with regard to PHC, after the professional who completed the course returns to their municipality of origin or allocation).

All analysis were performed at Stata v.14 (College Station, TX, EUA). Following Brazilian National Council Ethics (CEP) resolution 466/12, the work was submitted by Ethics committee in Federal University of Mato Grosso do Sul approval number CAAE 58735316400000021.

## Results

For the criteria listed, coverage by high and intermediate proportions of professionals who completed the CEABSF course exhibited an effect for some indicators.

Table 1 shows the mean and standard deviation distribution of professional graduates in CEABSF. In 2015, the State of Mato Grosso do Sul had a mean of 43 graduated professionals/per city. In the same year, it had a mean of 229,30 professionals in PHC/ per city (including physician, dentists, nurses and related professionals). The mean and confidence interval (CI95%) of each indicator (1 to 13) could be seen per year (2009 to 2015).

A lower rate of hospitalizations for primary care-sensitive conditions was seen; in other words, the number of hospitalizations decreased during the study period when related to an intermediate and high proportion of professionals who completed the course (Table 2, indicators 1, 2, 3, and 4). Likewise, the rates of diabetic patients registered and monitored (Table 1, indicators 7 and 8) and hypertensive patients enrolled and followed by these professionals were seen to increase (Table 2, indicators 9 and 10).

**Table 2:** Indicators related to hospitalizations for primary care-sensitive causes (Mato Grosso do Sul, 2009–2015).

Indicator 1—Hospitalizations for infectious gastroenteritis and complications			
	RR	CI95%	
T1 (low)	1.00		
T2 (intermediate)	0.96	0.85	1.08
T3 (high)	0.82	0.69	0.98
Indicator 2—Hospitalizations for asthma			
T1 (low)	1.00		
T2 (intermediate)	0.94	0.75	1.19
T3 (high)	0.67	0.48	0.93
Indicator 3—Hospitalizations for heart failure			
T1 (low)	1.00		
T2 (intermediate)	0.87	0.71	1.06
T3 (high)	0.73	0.55	0.95
Indicator 4—Hospitalization for primary care-sensitive conditions			
T1 (low)	1.00		
T2 (intermediate)	0.97	0.92	1.03
T3 (high)	0.91	0.84	0.99
Indicator 5—Care for diabetics			
T1 (low)	1.00		
T2 (intermediate)	1.12	1.00	1.25
T3 (high)	1.15	0.98	1.35
Indicator 6—Care for hypertensive patients			
T1 (low)	1.00		
T2 (intermediate)	1.12	1.01	1.25
T3 (high)	0.98	0.84	1.15
Indicator 7—Registration of diabetics			
T1 (low)	1.00		
T2 (intermediate)	1.24	1.13	1.36
T3 (high)	1.30	1.15	1.48
Indicator 8—Monitoring of diabetics			
T1 (low)	1.00		
T2 (intermediate)	1.22	1.11	1.34
T3 (high)	1.27	1.12	1.44
Indicator 9—Registration of hypertensive patients			
T1 (low)	1.00		
T2 (intermediate)	1.25	1.15	1.36
T3 (high)	1.26	1.12	1.42
Indicator 10—Monitoring of hypertensive patients			
T1 (low)	1.00		
T2 (intermediate)	1.23	1.13	1.34
T3 (high)	1.24	1.10	1.39
Indicator 11—Reported cases of workplace accidents			
T1 (low)	1.00		
T2 (intermediate)	1.13	0.94	1.36
T3 (high)	1.12	0.87	1.43
Indicator 12—Number of cancer deaths			
T1 (low)	1.00		
T2 (intermediate)	0.96	0.89	1.02
T3 (high)	1.01	0.92	1.12
Indicator 13—Deaths from traffic accidents			
T1 (low)	1.00		
T2 (intermediate)	1.07	0.95	1.22
T3 (high)	1.01	0.85	1.21

An effect was observed in (T_2_:intermediate) group, with an increase when analyzing care provided to diabetic patients (Table 2, indicator 5) and to hypertensive patients (indicator 6). These data confirm the benefits translated to the population which received primary care.

## Discussion

This research demonstrates that investment in Continuous Professional Development has a positive impact on reducing rates of hospitalization of PHC patients as well as improvement in management and monitoring indicators for chronic conditions.

Consequently, we believe that continuing professional education involving PHC teams should be encouraged, considering that they expand potential and helps to reduce hospitalizations for sensitive conditions [2], and also may improve other health indicators related to the PHC, specifically chronic conditions related to longitudinally and coordination of care. If the APS teams do not regularly accompany these conditions, hospitalizations for these sensitive conditions increase, creating a repetitive cycle that does not resolve the demands of community health. This may result, for example, in the increased need for admissions for sequelae of immune-preventable diseases and for the worsening of chronic conditions [23].

Although it may not be possible to isolate the effects of the CEABSF course in relation to the reduction in hospitalizations for primary care-sensitive conditions and improvement in indicators of chronic conditions, there is a connection evidenced in the results for asthma, gastroenteritis, and heart failure, as well as for registration and following of diabetic and hypertensive patients. The overall rate of hospitalization for primary care sensitive conditions did fall, and although specific rates only were seen to drop for 3 of these 19 conditions, these findings are potentially important considering that heart failure is the main isolated cause of hospitalizations for primary care-sensitive conditions in Brazil [30]. Furthermore, asthma and gastroenteritis rank alongside heart failure and bacterial pneumonia as the four main causes of hospitalization, accounting for approximately 80% of all hospitalizations for primary care-sensitive conditions in Brazil [30]. Because hospitalizations are among the main determinants of health costs, identifying impacts on the rates of these types of hospitalizations is essential [1].

These results may be related to the performance of professionals who are trained to carry out well-organized care activities, avoiding unnecessary hospitalizations and aggravation of illness to a stage that requires hospitalization [31]. The literature points to the importance of the training PHC professionals to obtain better rates of hospitalization for primary care-sensitive conditions [1,2]. However, few studies have investigated the relationship between better health indicators and the presence of specifically trained professionals on PHC teams. We were able to find only one study in the literature that showed an association between lower rates of hospitalization for primary care-sensitive conditions in patients attended by PHC teams which included medical specialists in family and community health, in comparison to teams with doctors without this expertise [19].

The results of this study are relevant since they demonstrate the transforming role of CPD, considering the contributions made by the professionals who completed the CEABSF training. The CPD impact the social context and quality of the PHC provided [2,19,32]. This is important, since PHC must be organized according to the social context, which is marked by intense inequality and the population within the territory to which the team is assigned [2].

In this arrangement, trained health professionals who are attentive to the health-disease process in the local community (in terms of diversity and inequity) and work with the real demands of the population can provide adequate specific care in the context of PHC that plays an important role in improving quality of care and boosting the impact of care [19,32].

Cases like the CEABSF course, namely graduate-level training for professionals in FHS guided by CPD principles, epidemiology, and sociodemographic data, can develop and strengthen professional skills so that team members can serve, respond, and be responsible for community health needs [33]. In the context of health, PHC work has specific characteristics that require specialized graduate training [2]; it is the only care scenario which serves patients of all ages and sexes to treat the most common conditions, considering the context of patient families and the community [33].

In this way, the training model of the UNA-SUS initiative is in line with the principles of FHS, by offering contextualized curriculum to meet the bespoke needs of the region and the realities of practice in the community [22]. The training process developed according to the foundations of CPD provides an essential foundation and a broader view of the daily challenges faced by health professionals [34]. The guiding axis of CPD lies in daily practice and its importance as a source of knowledge, considering that practices are influenced by multiple factors. The objective is the transformation of praxis, using participatory training strategies and considering the specifics of each context, and also emphasizes multiprofessional contributions and interdisciplinarity [35,36,37].

Because in Brazil, control and prevention of chronic conditions (especially diabetes and hypertension) are the responsibility of PHC teams [38], the CEABSF course analyzed herein prioritized this line of care to stimulate reflective thinking and teamwork in order to confront these diseases which are so common in the general population. Several aspects were addressed, but the one which may have had the strongest influence was the increase in registration and follow-up of hypertensive and diabetic patients, which stimulated organization and planning of the working process in the multiprofessional team. This indicates the possibility of greater control of these diseases over the medium and long term, and can lead to reductions in related morbidity and mortality, which pose a great challenge for PHC [38]. Furthermore, expanded monitoring of these chronically ill patients augments longitudinality of care, treatment of injuries, and interventions addressing risk factors in order to improve health, prevent or reduce complications, and reduce the costs of hospitalizations [39]. The impact on management of these chronic conditions through an organized work process which involves the population can make it easier to confront determinant and conditioning factors in the health-disease process, and may be effective in stimulating self-care and autonomy in individuals with diabetes and hypertension [38].

The results demonstrate that the course had a positive impact on the care of diabetics and hypertensives (care, registration and monitoring). The improvement obtained through educational processes involving multidisciplinary teams was considered the second most frequent strategy to increase access to diabetes care in PHC; the most frequent strategy was reorganization of services [39]. This reinforces the conviction that the continuous education process can produce significant changes in health care activities that may affect the development of complex diseases (with regard to etiology, pathogenesis, and management/prevention) which in turn may require more complex services, specialized care, and higher cost when they are not addressed within the scope of PHC.

Considering that registration and follow-up of hypertensive and diabetic patients has increased in territories where CEABSF graduates work, we can infer that longitudinality of care is being included in these areas. This is important, because it denotes a long-term relationship between health professionals and their patients and promoting a broader reach for PHC, since this becomes these people’s regular source of directed care. In the medium and long term, greater closeness between users and the service in the longitudinal process of care can result in lower utilization of services, better preventive care, decreases in preventable diseases, easier recognition of specific problems in each community/patient, fewer hospitalizations, and lower total costs of health services [40].

One of the strengths of this study lies in the fact that we adopted comparative parameters (traffic accidents and workplace accidents) to contrast with indicators related to PHC. The results for these indicators were not impacted by the presence of course graduates, demonstrating that the statistical modeling selected was adequate for the type and object of study. Another strength was the analysis of indicators over seven years, long enough for changes in practices among the FHS teams to contribute to the improved results found in the health indicators where these course graduates work.

The present study has some limitations. Data from a secondary database requires caution, since it may present limitations related to scope and quality. Population data from the 2010 Brazilian Census were used [29], as well as information about the number of hospitalizations for the period 2009–2015. The data on hospitalization for primary care-sensitive causes does not detail whether patients participated in FHS. Finally, hospital availability in the SUS system during the period under study may affect hospitalization rates, but was not included in the analysis. Despite these limitations, to our knowledge this is the first study to assess and demonstrated the impact of a PHC specialization course on health outcomes in the context of a Brazilian Midwest state.

These results need to be corroborated by investigations that reduce or overcome the limitations mentioned above, and evaluating quality aspects of services through validated instruments for PHC, such as PCATool [41]. New studies are recommended in other Brazilian states where specialization courses in family health have been held via UNA-SUS, and to evaluate other CPD initiatives for PHC.

## Conclusions

The course impacted important indicators related to the attributions of PHC professionals, considering that reduced rates of hospitalization for primary care-sensitive conditions (overall rate of sensitive conditions, and specific rates for asthma, gastroenteritis, and heart failure) were seen in the territories where professionals who completed this course, along with increased registration and monitoring of diabetic and hypertensive patients. New formulations for PHC training courses could be implemented, highlighting the CPD’s contribution to improving the performance of health services. Thus, investments and training offers to FHT professionals must be part of the priority goals for health management in the country.

## Supporting information

S1 Dataset (XLSX)
